# Assessment of monoclonal antibody glycosylation: a comparative study using HRMS, NMR, and HILIC-FLD

**DOI:** 10.1007/s00216-024-05261-5

**Published:** 2024-04-06

**Authors:** Joshua Shipman, Michael Karfunkle, Hongbin Zhu, You Zhuo, Kang Chen, Milani Patabandige, Di Wu, Mercy Oyugi, Richard Kerr, Kui Yang, Sarah Rogstad

**Affiliations:** 1 Division of Complex Drug Analysis, Office of Testing and Research, Center for Drug Evaluation and Research, US Food and Drug Administration, St Louis, MO 63110 USA; 2 Division of Pharmaceutical Analysis, Office of Testing and Research, Center for Drug Evaluation and Research, US Food and Drug Administration, St Louis, MO 63110 USA; 3 Immediate Office, Office of Testing and Research, Center for Drug Evaluation and Research, US Food and Drug Administration, Silver Spring, MD 20993 USA; 4 Present Address: Office of Biotechnology Products, Center for Drug Evaluation and Research, US Food and Drug Administration, Silver Spring, MD 20903 USA; 5 Present Address: Center for Devices and Radiological Health, US Food and Drug Administration, Silver Spring, MD 20903 USA; 6 Present Address: AbbVie, South San Francisco, San Francisco, CA 94080 USA; 7 Present Address: Sanofi, Framingham, MA 01701 USA

**Keywords:** Mass spectrometry, Multi-attribute method, N-Glycan analysis, Monoclonal antibody, Nuclear magnetic resonance

## Abstract

Monoclonal antibodies (mAbs) represent the largest class of therapeutic protein drug products. mAb glycosylation produces a heterogeneous, analytically challenging distribution of glycoforms that typically should be adequately characterized because glycosylation-based product quality attributes (PQAs) can impact product quality, immunogenicity, and efficacy. In this study, two products were compared using a panel of analytical methods. Two high-resolution mass spectrometry (HRMS) workflows were used to analyze N-glycans, while nuclear magnetic resonance (NMR) was used to generate monosaccharide fingerprints. These state-of-the-art techniques were compared to conventional analysis using hydrophilic interaction chromatography (HILIC) coupled with fluorescence detection (FLD). The advantages and disadvantages of each method are discussed along with a comparison of the identified glycan distributions. The results demonstrated agreement across all methods for major glycoforms, demonstrating how confidence in glycan characterization is increased by combining orthogonal analytical methodologies. The full panel of methods used represents a diverse toolbox that can be selected from based on the needs for a specific product or analysis.

**Graphical Abstract:**

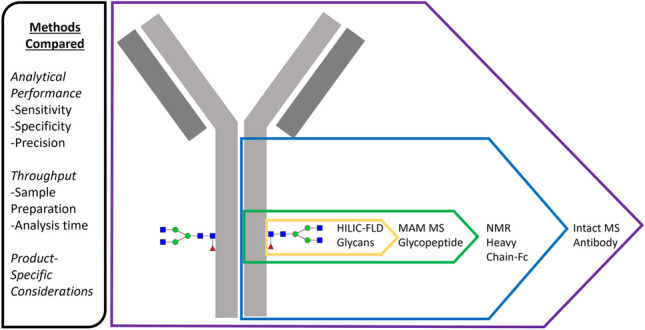

**Supplementary Information:**

The online version contains supplementary material available at 10.1007/s00216-024-05261-5.

## Introduction

Analytical characterization of the structure and chemistry of monoclonal antibodies (mAbs) in a formulation is critical for quality assessment of these products. In particular, glycosylation, the enzymatic attachment of oligosaccharides to the protein backbone, is a critical quality attribute (CQA) that should be characterized and controlled. Glycan composition can depend on many variables including product type, expression system, and cell culture conditions [1, 2]. Different glycan distributions can impact the quality, safety and efficacy of mAbs and other therapeutic protein drugs [3–8]. Analytical techniques play an important role in glycan characterization and can be used to detect product differences that may not be identified by pharmacokinetic (PK) profiling. Analytical comparability between products also must be demonstrated for a proposed biosimilar to meet the standards for approval [9, 10].

N-glycans, oligosaccharides connected to proteins at asparagine (N) residues, can differ in monosaccharide composition, branching, and connectivity which makes their analysis challenging. The conventional workflow for N-glycan analysis includes enzymatic release from the protein followed by labeling of the reducing end aldehyde with a fluorophore such as 2-aminobenzamide (2-AB) or 2-aminobenzoic acid (2-AA) [11]. Glycans can then be separated by hydrophilic interaction chromatography (HILIC) and detected and quantified with fluorescence detection (FLD). This general approach is widely used for quality control testing of glycans in therapeutic proteins. In addition to released glycan approaches, mass spectrometry-based methods are also widely used in the characterization section of therapeutic protein biologics license applications (BLAs) [12]. In recent years (2016–2020), mass spectrometry (MS) was found to be used for characterization of released glycans in nearly 70% of all BLAs [13].

High-resolution techniques, such as MS, offer the advantage of increased precision when compared to some conventional methods. This study focused on benchmarking high-resolution mass spectrometry (HRMS) and nuclear magnetic resonance (NMR) techniques using state-of-the-art instrumentation against conventional HILIC-FLD quantitation. Glycans can be analyzed via HRMS using a variety of workflows. MS detection can be performed parallel to FLD of released glycans offering mass-confirmation of glycan species along with improved sensitivity and specificity; additionally, modern labeling reagents such as RapiFluor-MS (RFMS) have been engineered which provide reduced sample preparation times and improved MS performance [11, 14]. Proteolytic digestion of the proteins to produce glycopeptides can be performed to provide site-specific information by analyzing mAbs at the peptide level, which also allows for the characterization of other CQAs such as oxidation and deamidation simultaneously. One LC-MS-based analysis approach that can be applied to glycopeptides is known as the multi-attribute method (MAM). The biopharmaceutical industry has shown increasing interest in using this approach in quality by design (QbD) and quality control (QC) roles [15, 16]. Modern mass spectrometers offering resolutions greater than 100,000 in combination with deconvolution software can be used to analyze intact or partially digested proteins. These types of analyses offer minimal sample preparation compared to the other approaches discussed and can thus result in fewer artifacts.

A middle-down NMR method has been developed to separate Fc and Fab domains from intact mAb first, followed by urea denaturation of Fc domain to profile intact N-glycan distributions. In NMR spectra, each glycan monosaccharide peak was identified and quantified to indirectly inform the intact glycan distribution profile [17, 18]. As a result, this middle-down NMR approach monitors monosaccharide content (e.g., galactosylation) instead of specific glycan moieties (e.g., FA2) and cannot be directly compared to the LC- and MS-based method results.

**Fig. 1:**
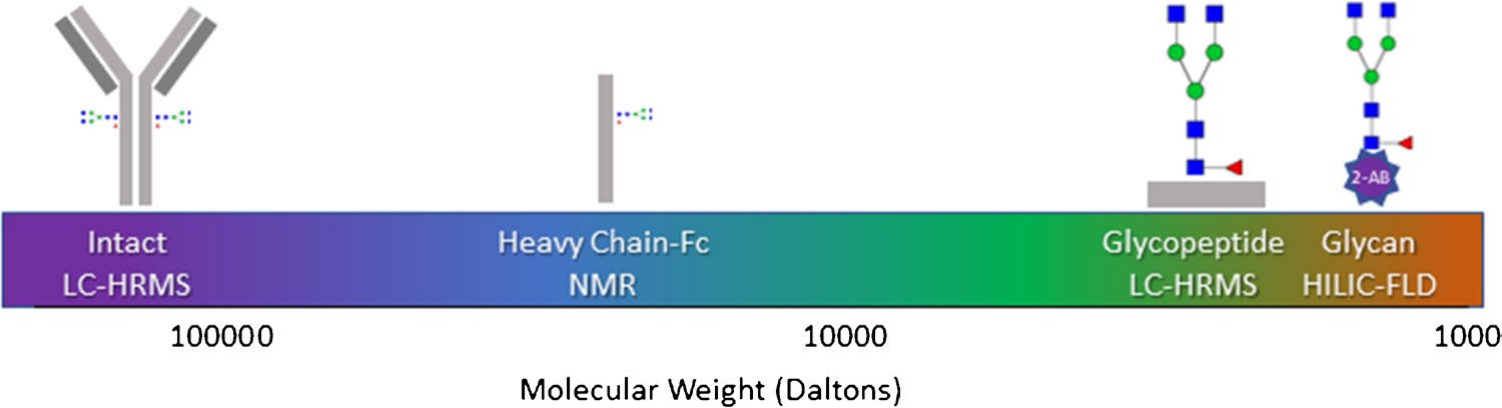
Overview of approaches. The methods assessed in this study were used to analyze the glycan distribution of a mAb using a range of digested species. The analytes and methods used within the study are depicted along a molecular weight scale

In this study, detailed analytical characterization using multiple orthogonal analytical techniques was performed including analysis by HILIC-FLD of released glycans, MAM, intact mass LC-MS, and middle down NMR. Figure 1 presents an overview of the approaches used along with the size of the relevant analytes. Rituximab was used as a model mAb. Products were analyzed from two sources, one approved and one not approved for the US marketplace by the FDA. The ability of each method to identify differences between the two products was assessed, an important aspect to consider as firms begin to introduce more biosimilars into the marketplace which must be properly characterized to demonstrate a highly similar profile to the reference product prior to approval. Additionally, advantages and disadvantages of each approach were considered.

## Methods

### Samples

Nine lots (18 vials) of rituximab drug product from two different manufacturers, including product A, the FDA approved innovator product Rituxan (Genentech), and product B, the foreign-sourced FDA unapproved product Reditux (Dr Reddy’s). For product A, seven lots were analyzed (denoted as products A1–A7), and for product B, two lots were analyzed (denoted as products B1–B2) based on lot availability.

### Released glycan analysis by HILIC-FLD

Rituximab samples were analyzed using a HILIC-FLD method. 2-AB labeled N-glycans were prepared using a 2-AB Kit (Proenzyme, Signal 2-AB-plus Labeling Kit) according to the manufacturer’s recommended protocol. Briefly, samples were buffer exchanged with digestion buffer using a 10-KDa MWCO filter (PALL Corporation, Ref OD010C33). N-Glycans were labeled with the 2-AB labeling reagent after they were cleaved by PNGase F (New England BioLabs, P0704L, 500 000 U/mL) and purified from protein counterparts by the MWCO 10K Da filter. The N-glycans were further purified using a HILIC SPE cartridge (HyperSep-96-Diol, Thermo-Fisher 60300-630), for full labeling protocol see Appendix S1. The N-Glycans were separated by a HILIC column (Waters Acquity BEH Amide cloumn, 2.1 × 150 mm, 1.7 µm, 130 Å.). The labeled glycans were detected by fluorescence detector with an excitation wavelength of 330 nm and an emission wavelength of 420 nm. The relative peak area of each individual N-glycan peak to the total N-glycan peak area was used for the quantitation of released N-glycan. Samples were analyzed with duplicate injections of duplicate preparations (*N*=4). HILIC-FLD experimental parameters were adapted from a Waters application note [19]. Additional methodological details can be found in Table S1.

### Glycopeptide analysis by multi-attribute method (MAM)

Rituximab lots were digested with trypsin as previously described [20]. Fifty micrograms of rituximab was used for each processing replicate. Each product was reduced in 10 mM dithiothreitol (DTT) in 7.5M GdnHCl for 30 min at room temperature. The reduced protein was alkylated with 20 mM iodoacetic acid (IAA) for 20 min at room temperature in the dark. The reaction was quenched with an additional 10 mM DTT. Samples were desalted using 100 mM ammonium bicarbonate buffer and Zeba spin 7K MWCO 0.5 mL desalting columns. Samples were loaded on top of the Zeba column resin after removal of column storage solution and three rounds of column equilibrium with 100 mM ammonium bicarbonate. After loading samples, an additional 15 μL stacker of 100 mM ammonium bicarbonate was added on top of the gel bed to ensure maximal protein recovery. One microgram per microliter of trypsin was added at a 1:10 (enzyme/substrate ratio) and incubated for 30 min at 37 °C. Lastly, 3 μL of formic acid was used to quench the digest.

The nine rituximab lots were analyzed in triplicate (*N*=3, 5 μg per injection). Separated samples were analyzed on a Thermo Q Exactive hybrid quadrupole-Orbitrap mass spectrometer with a HESI source. MAM LC-MS experimental parameters are available in Table S2. Product quality attributes (PQAs) including glycosylation were analyzed using Thermo Chromeleon 7.2. PQAs were defined using retention times and theoretical *m/z* values. Relative abundance levels of PQAs were calculated using the area under the curve of *m/z* peaks, where the area of the modified peak was compared to the total area for the modified and unmodified peaks.

### Glycoprotein analysis by intact mass LC-MS

Reverse-phase (RP) LC-MS was used to identify, with accurate mass information, the predominant post-translationally modified (PTM) variants of rituximab without the use of proteolytic digestion. Samples were analyzed with triplicate injections of triplicate preparations (*N*=9).

Ten-kilodalton molecular weight cut-off (MWCO) filters were washed three times using 95:5 water/acetonitrile (ACN) with 0.1% formic acid, centrifuging at 10,000 g for 5 min, and then loaded with 20 µg of sample. Samples were buffer exchanged with three 500 µL volumes of 95:5 water/ACN with 0.1% formic acid, centrifuging 5 min at 10,000 g twice and 10 min at 10,000 g once. Recovered sample was diluted to a final concentration of 0.20 mg/mL in 95:5 water/ACN with 0.1% formic acid. Intact LC-MS experimental parameters are available in Table S3 [21].

Data were processed using BioPharmaFinder version 4.0, the Intact Mass Analysis workflow. The spectrum was averaged over the retention time range of 5.5 to 8.5 min and deconvoluted with the ReSpect™ deconvolution algorithm.

### Glycosylation analysis by middle-down NMR

A published middle-down NMR method [17, 18] was followed to prepare the rituximab fragment crystallizable (Fc) samples. NMR spectra were obtained on a Bruker Ascend 850 MHz spectrometer equipped with a cryogenic TCI probe. A modification of Bruker ^1^H-^13^C heteronuclear single quantum coherence (HSQC) pulse hsqcetgpsi2 was used [18]. Briefly, the total acquisition time was 10.5 h with 1024 complex data points for the ^13^C dimension and 24 scans averaged for each free induction decay (FID). The ^13^C spectral width was 100 ppm, and the carrier was set at 95 ppm. The data were processed using MestReNova 11.0.3. Each FID was apodized with 90° sine square function, and the first data point was scaled to half. Zero filling was up to 4096 points in the ^1^H dimension and 2048 points in the ^13^C dimension. The spectra baseline correction was performed using a third-order polynomial fit. ^1^H chemical shift values were referenced to internal trimethylsilylpropanoic acid (TSP), and ^13^C chemical shifts were calculated based on ^1^H of TSP. Peak intensity was read out using MestReNova 11.0.3.

## Results/discussion

To assess and compare analytical methods for the quantitation of glycans, multiple approaches were conducted using approved and unapproved rituximab as a model mAb. Results were assessed individually and compared across methods. The glycans in this study were reported using Oxford notation; a table of the glycan structures and nomenclature can be found in Table S4 [22].

### Released glycan analysis by HILIC-FLD

**Fig. 2:**
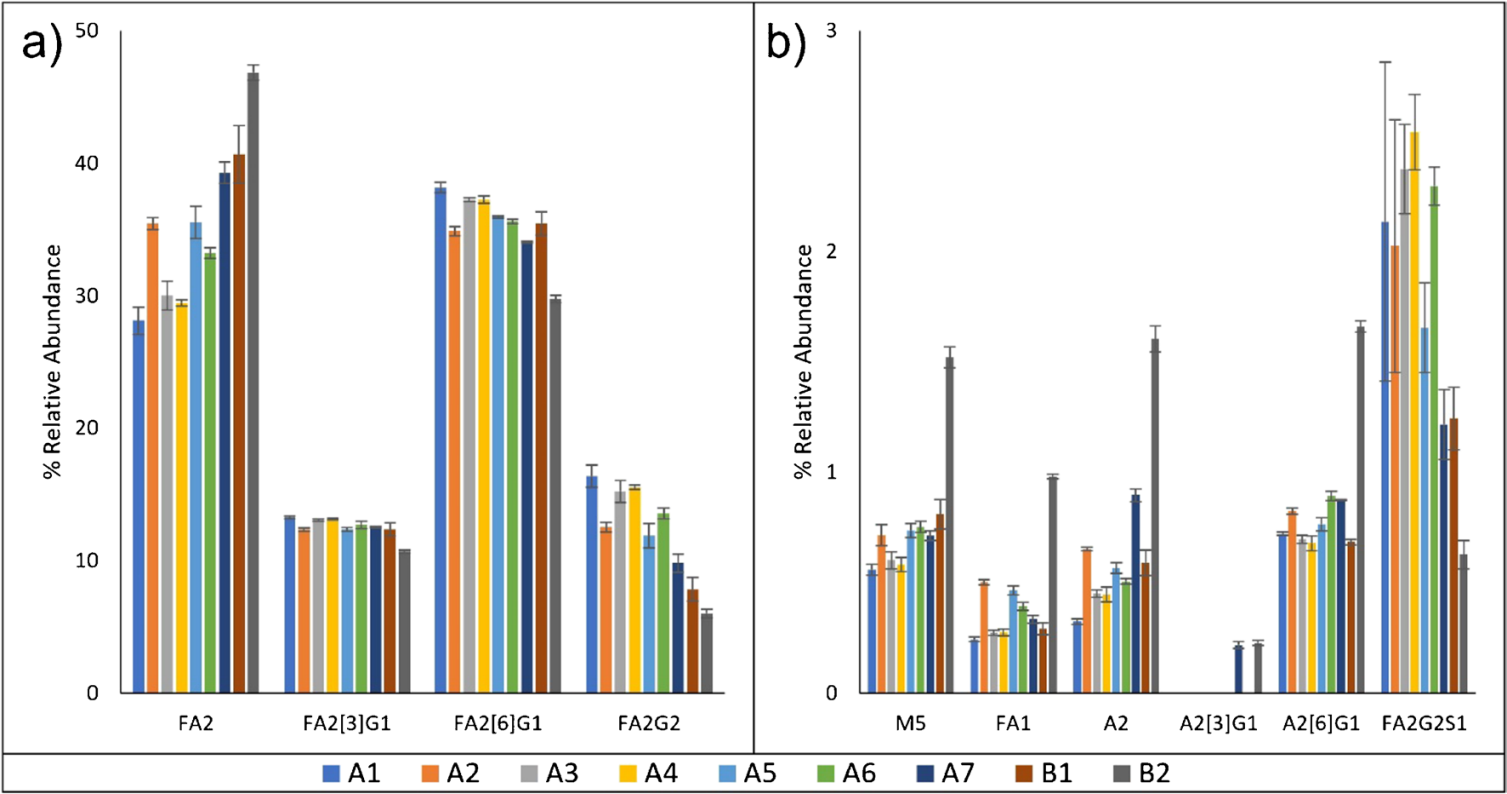
HILIC-FLD results. Twelve glycan species were identified by HILIC-FLD, including branched isomers. **a** High abundance glycans, **b** low abundance glycans. Lots shown include products A1–A7 and products B1–B2. *N*=4; error bars represent standard deviation

A HILIC-UHPLC with FLD detection method was applied to determine the relative glycan distribution of the nine lots of rituximab. *N*-glycans were identified with the aid of reference chromatograms. Figure 2 shows the distribution of 2-AB labeled Rituximab *N*-glycans derived from products A and B; representative chromatograms can be found in Figure S1. The limit of detection for FLD was 0.2%; this value was determined comparing signal to a blank in accordance with ICH guidance for validation of analytical procedures [23, 24].

A total of 12 *N-*glycans from rituximab samples were identified by comparison to reference data [25]. The N-glycans in rituximab samples were labeled with 2-AB labeling reagent after they were cleaved and purified from protein counterparts and analyzed. Quantification of the *N*-glycan profiles was also performed for each of the rituximab drug products. In all rituximab samples analyzed, four major glycans were identified with more than 5% relative abundance. The relative distribution of FA2[3]G1 and FA2[6]G1 were found to be more consistent than other glycans between manufacturers, and within the same manufacturer among different lots, as shown in Table S5 and Fig. 2. For product B, only two lots (B1 and B2) were analyzed in this study. These two lots from product B showed obvious variances in medium and low abundant glycans. In addition, FA2 and FA2G2 were identified as the two major glycans that demonstrated a difference in the glycan profiles between the drug products.

### Glycopeptide analysis by multi-attribute method (MAM)

MAM is a peptide mapping method where mAbs (or other proteins) are enzymatically digested, then separated and detected with LC-MS. MAM can simultaneously identify and quantify multiple PQAs in therapeutic proteins and provides information at the molecular level that may not be present in conventional QC methods. In general, MS-based peptide mapping techniques were used for characterization in 100% of BLAs from 2016 to 2020 [9]. However, MAM has been specifically identified in very few regulatory applications, despite its prevalence in the literature.

**Fig. 3:**
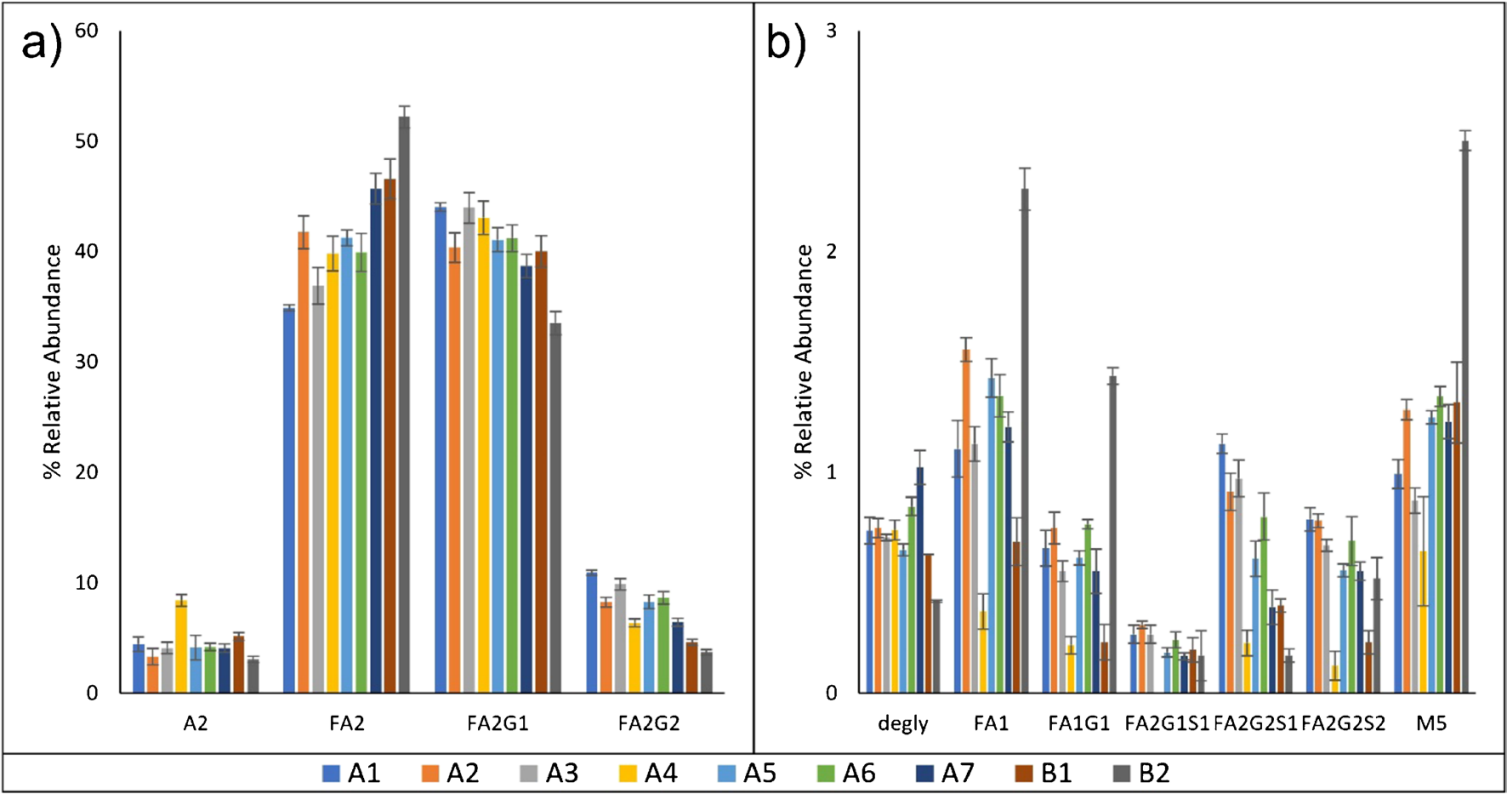
MAM results. MAM was used to identify 11 glycopeptides, including the deglycosylated species. **a** High abundance glycans, **b** low abundance glycans. Lots shown include products A1–A7 and product B1–B2. *N*=3; error bars represent standard deviation

A MAM approach was developed and validated in-house to quantify glycosylation, oxidation, deamidation, lysine clipping, and N-terminal pyroglutamate in rituximab [20]. The nine lots of rituximab drug product for the current study were analyzed by MAM, which detected and quantified 21 PQAs. Figure S2 shows an example of the total ion chromatograms (TICs) of products A and B with identified tryptic peptides (Table S6). The raw data were first searched with Proteome Discoverer against a database containing the rituximab protein sequence to map peptides and modifications. Ten glycopeptides as well as the deglycosylated peptide were detected using this method. The relative abundance levels of the monitored glycopeptides can be found in Fig. 3 and Table S7. During method validation, the LOQ for peptide detection was determined to be 0.036 pmol, or 0.1% fractional abundance, using a calibration curve approach. No significant differences were detected in glycan abundance between products A and B. However, significant differences were found for multiple low abundance glycans for lot B2 alone. These lot differences are similar to those found using FLD.

### Glycoprotein analysis by intact mass LC-MS

**Fig. 4:**
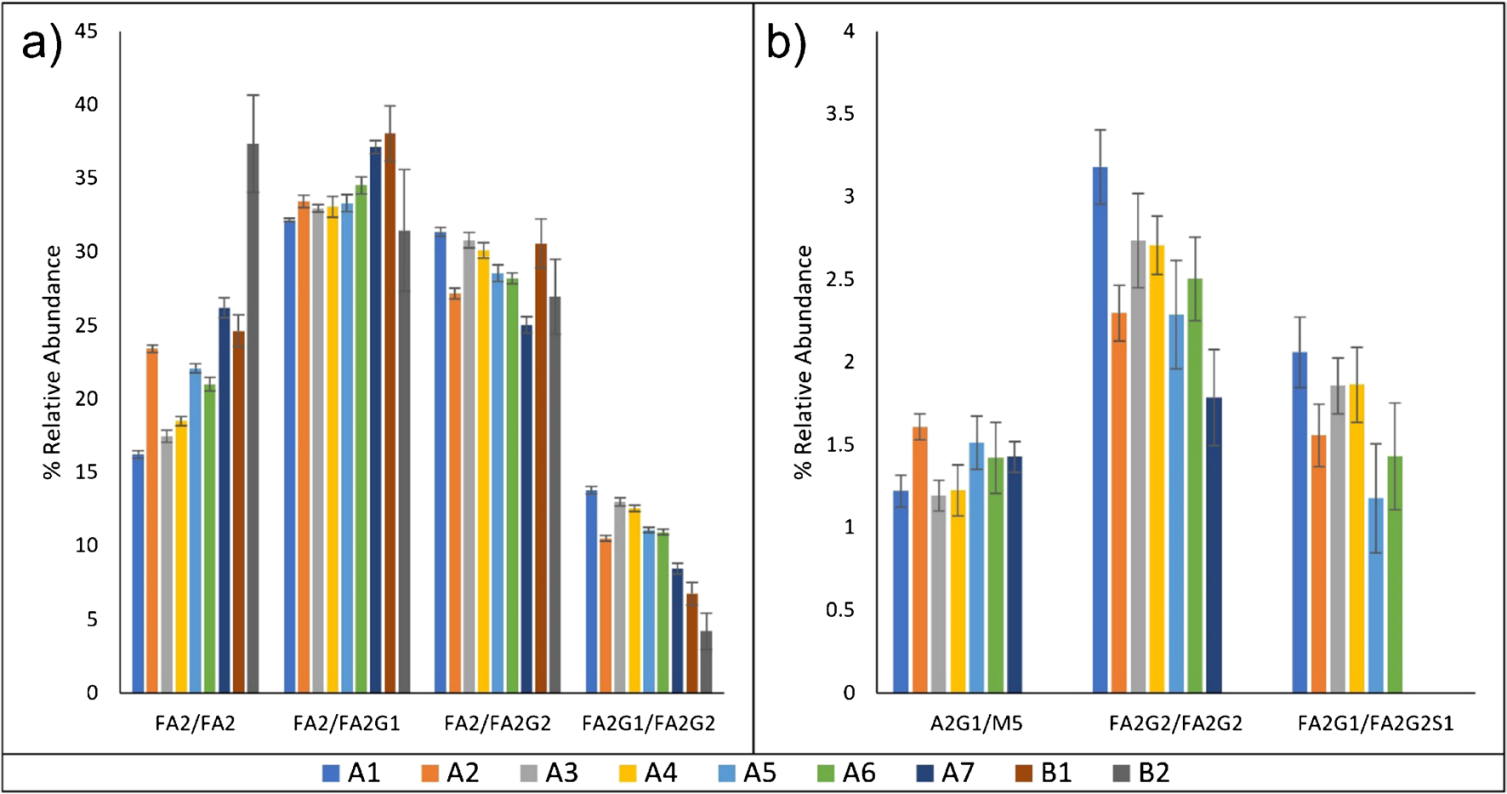
Intact mass analysis results. Intact mass analysis was used to identify eight protein glycoforms. **a** High abundance glycans, **b** low abundance glycans. Lots shown include products A1–A7 and products B1–B2 (sum of all clipping variants). *N*=9; error bars represent standard deviation

Intact mass measurements provide simple, rapid measurements with minimal sample preparation. Each species identified by intact mass measurement represents the two glycans on a given mAb. Different isobaric combinations can result in ambiguous assignments; for example, a theoretical mass of 147,399 Da corresponds to two glycan pairings: FA2G0/FA2G2 and FA2G1/FA2G1, one of the three most abundant species in every lot tested. Glycan assignments were made under the assumption that glycosylation in mAbs is primarily composed of biantennary complex glycans, proteins which have more branched or complex-type glycans and there will have more isobaric combinations. Seven glycoforms were identified by intact mass measurement (Fig. 4 and Table S8); five of these were combinations of the FA2/FA2G1/FA2G2 glycans, while one glycoform contained a sialylated glycan (FA2G1/FA2G2S1), and one contained a high-mannose glycan (A2G1/M5). The reproducibility for intact analysis was comparable to other methods; however, the method had comparatively worse sensitivity. Exact limits of detection were not determined. However, masses with a fractional abundance below 1% in product A and 2% in product B could not be reproducibly interpreted by the deconvolution algorithm, and these values were used as approximations of the LOQs. These masses were therefore excluded from analysis. Interestingly, it was found that the lower sensitivity in product B was the result of incomplete C-terminal clipping, which resulted in a greater degree of spectral complexity as the glycan populations were split between antibodies with 0-2 lysine residues. Representative spectra are shown in Figures S3. More complex mAbs such as those with multiple glycosylation sites may not be suited for intact analysis, for example, the mAb cetuximab which contains a Fab glycan in addition to the conserved Fc glycosylation site which has been analyzed using a reduced analysis [26]. RPLC-MS analysis offered poor chromatographic separation of glycoforms and sensitivity could be increased using orthogonal separation techniques including HILIC-MS, SEC-MS, or CZE-MS [27, 28].

### Glycosylation analysis by middle-down NMR

Glycan analysis by middle-down NMR quantifies individual monosaccharides by measuring the unique chemical shifts specific to the anomeric C-H groups of glycans. The measured ^1^H-^13^C HSQC spectra represent a molecular fingerprint that is highly reproducible between different mAb products and laboratories. The spectra can be used to profile the composition of N-glycans in mAbs and quantify epitopes which are known to effect biotherapeutic products [2, 9, 17, 18]. Specifically, anomeric signals representing individual monosaccharides were observed in the range of 4.4 ~ 5.3 ppm for ^1^H and 98 ~ 107 ppm for ^13^C in the HSQC spectrum, with the exception of GlcNAc1, at 5.1/81.2 ppm, due to its covalent linkage to asparagine.

**Fig. 5:**
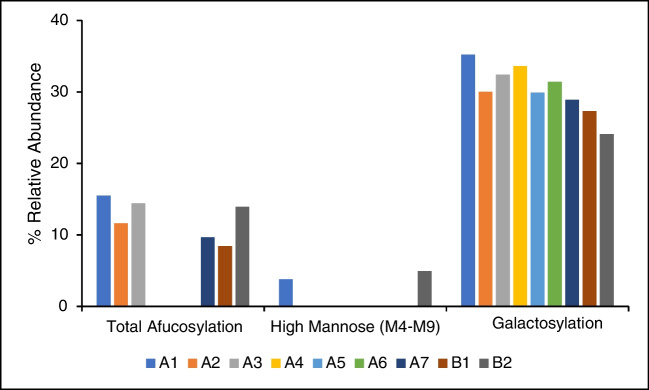
Quantification of glycans using NMR. Lots shown include products A1–A7 and products B1–B2. *N*=1; error bars represent 10% variation as determined by previous experiments due to low sample availability

The two drug products were characterized by HSQC, the anomeric region for product A is shown in Figure S4. The anomeric regions of the two rituximab products were compared quantitatively to characterize their *N*-glycan profiles; the LOQ for the NMR method was determined to be 1.5% for an individual terminal monosaccharide, as previously reported [17, 18]. Single replicates were measured for each lot of drug product. Precision can be estimated using previously published results which indicate the average CV for detected monosaccharides is 13% (*N*=6) [18]. Detection of the major N-glycan peaks (Fuc, GlcNAc2, Man3, Man4(3), Man4(6), GlcNAc5(3,T), GlcNAc5(6,T), and Gal6) indicated that complex forms of N-glycans could be identified and that they made up the predominant glycoforms. The cellulose peaks shown in both samples were contaminants from the sample dialysis process using cassettes. An unknown peak **a** was observed in both products. Another unknown peak **b**, close to fucose, was detected in product B only. Quantification using the peaks Man4, GlcNAc2, and GlcNAc5 was carried out using Eqs. 1–3 below. The results are shown in Fig. 5 and Table S9. The afucosylation levels were low in all samples, below 16%. The low signal to noise ratio (S/N) for GlcNAc2(aF) was the cause of large CVs of 21% and 35% in product A and product B, respectively; in some lots of product A GlcNAc2(aF) was not observed. ManB, mannose at position 5 of the 1-6/1-6 branch,^13^ as an indicator of high-mannose N-glycan, was of greater intensity visually in product B than in product A. The calculation of high-mannose species (Eq. 2) was only applicable for one lot in products A and B due to sensitivity issues. Finally, Gal% calculated by Eq. 3 was the percentage of galactosylation in the complex form only. GlcNAc5 C5-H5 does not differentiate branches. Therefore, 100% galactosylation meant that two galactose monosaccharides were present in one complex N-glycan with two branches. Pure A2G1 and A2G2 would have Gal% of 50% and 100%, respectively. In the given sample set, Gal% of product A was 30% more than that of product B, consistent with other results (Table 1). Sialylated species were not observed in either product A or B; results from other methods indicate these species are below the LOQ for NMR analysis.

**Table 1:** PQA measurement comparisons. Average relative abundance values of PQAs are shown for all methods across products. *NA* not applicable (one replicate), *ND* not detected

		Total afucosylation^a^			High-Mannose			Galactosylation			Sialylation		
		Average (%)	SD	CV	Average (%)	SD	CV	Average (%)	SD	CV	Average (%)	SD	CV
Product A	FLD	2.0	0.4	18	0.67	0.08	12	65	4	6	2.0	0.5	26
	MAM	6	2	27	1.1	0.3	24	51	4	7	1.5	0.6	40
	Intact	1.4	0.2	14	1.4	0.2	14	52	3	6	1.4	0.7	48
	NMR	13	3	20	3.8	NA	NA	32^b^	2	7	ND	ND	ND
Product B	FLD	4	1	41	1.2	0.4	31	53	5	9	0.9	0.3	34
	MAM	6.0	0.6	10	1.9	0.7	35	42	4	9	0.8	0.1	14
	Intact	ND	ND	ND	ND	ND	ND	42	4	11	ND	ND	ND
	NMR	11	4	35	4.9	NA	NA	26^b^	2	9	ND	ND	ND

^a^Total afucosylation includes both complex afucosylation and high-mannose species

^b^The calculation of Gal% using NMR reflected the occupancy of Gal at both 1-3 and 1-6 branches, i.e., A2G1 and A2G2 would be 50% and 100%, respectively

1 $$\mathrm T\mathrm o\mathrm t\mathrm a\mathrm l\;\mathrm{aF}\mathrm u\mathrm c\%\;={''}\mathrm{GlcNAc}2(\mathrm{aF}){''}\;/({''}\mathrm{GlcNAc}2(\mathrm{aF}){''}\;+\;{''}\mathrm{GlcNAc}2{''})\;\times\;100$$

2 $$(\mathrm M4-\mathrm M9)\%=(''\mathrm{Man}4(6,\mathrm{HM})'')/(''\mathrm{Man}4(6)''+''\mathrm{Man}4(6,\mathrm T)''+''\mathrm{Man}4(6,\mathrm{HM})'')\times100$$

3 $$\mathrm G\mathrm a\mathrm l\%=(''\mathrm{GlcNAc5}(\mathrm C5-\mathrm H5)'')/(''\mathrm{GlcNAc}5(\mathrm C5-\mathrm H5)''+''\mathrm{GlcNAc}5(\mathrm C5-\mathrm H5,\mathrm T)'')\times100$$

### Comparison of methods for N-glycan analysis

**Fig. 6:**
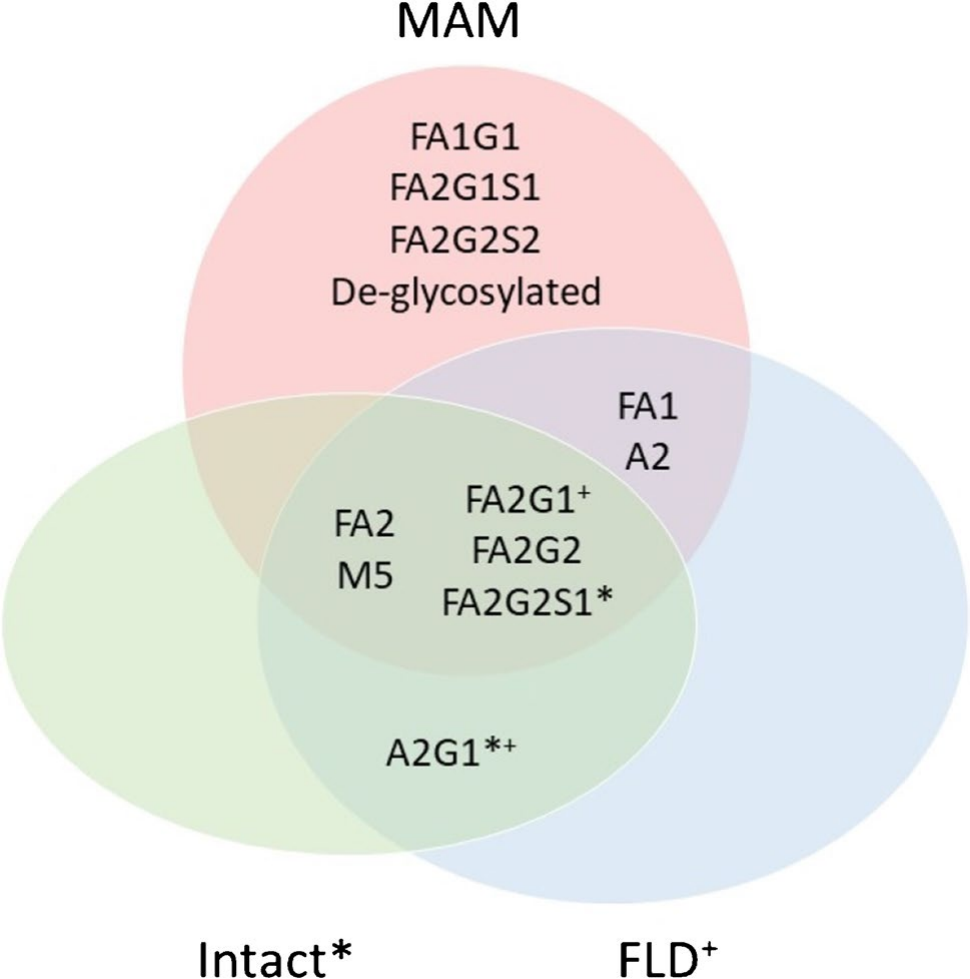
Venn diagram of identified glycan species. Identified glycan species are shown by method. * These identifications were inferred over other isobaric species based on data from other approaches. + Branched isomers of these species were identified using FLD, while other methods were unable to differentiate between isomers

The total number of glycan species identified varied between methods. A Venn diagram of species identified by each method is shown in Fig. 6. FLD analysis identified 12 unique glycoforms and was the only method that could differentiate between 1-3 and 1-6 linkages in the current study, though high-resolution NMR has demonstrated branch linkage differences in other studies [17, 18]. MAM identified 11 glycan species and was the only method that could quantitate the de-glycosylated species. MAM and FLD analysis were the only methods that identified unique glycoforms. Reduced analysis identified four glycans, while intact identified seven glycans. Intact analysis contained some isobaric combinations which were ambiguous. In some instances, assumptions were made about intact assignments based on data provided by other methods, for example, for the FA2G2S1/FA2G1, assignment was made instead of FA2G1S1/FA2G2 because the former were higher abundance species across the other methods.

**Fig. 7:**
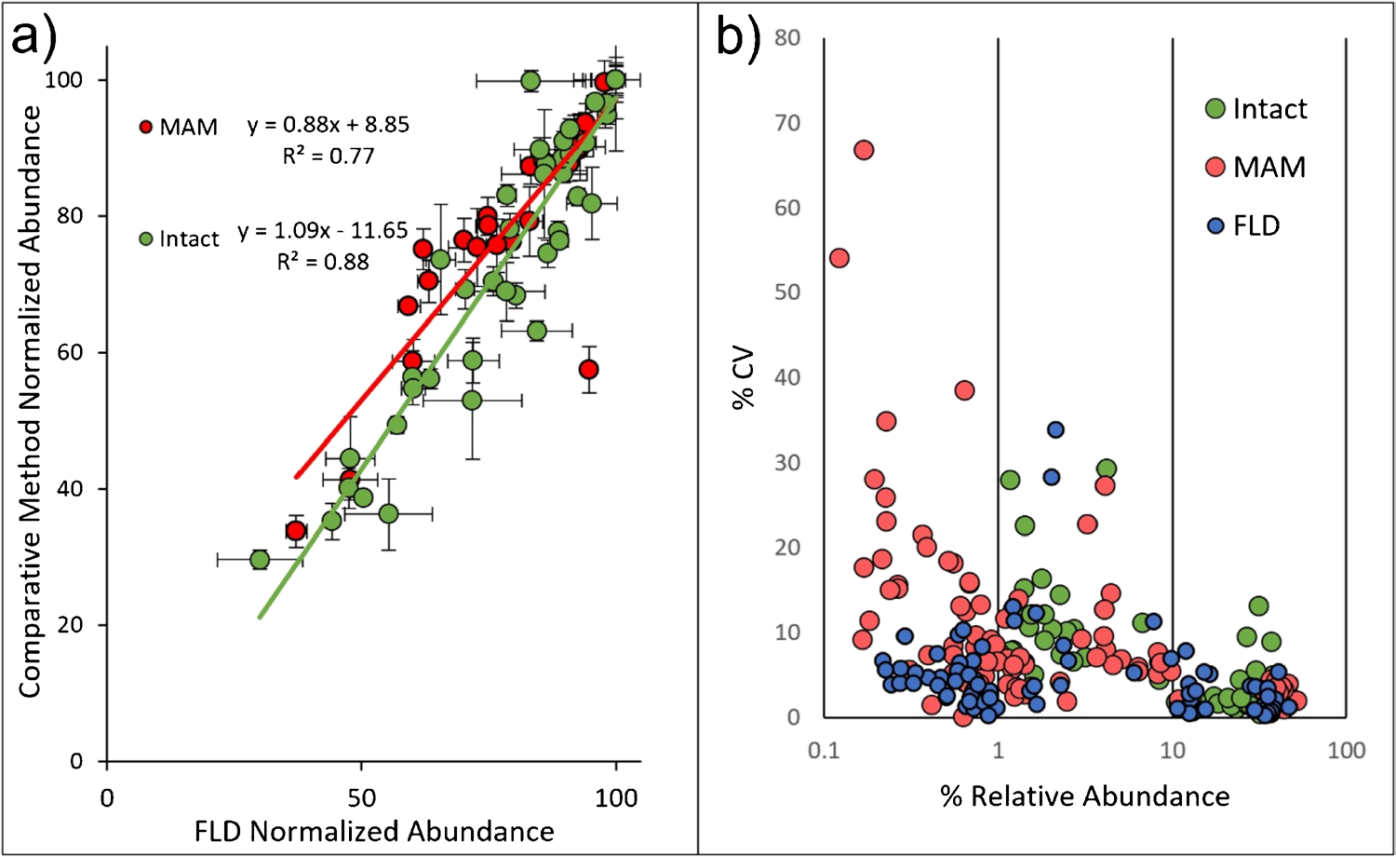
Comparison of glycan species. **a** Glycan features for individual MS methods were compared to FLD values. Linear trends were observed for both methods. Error bars represent standard deviation. **b** Coefficients of variation (%CV) for technical replicates of individual glycan species, number of injections varied by method

Similar trends were observed across lots for high abundance glycans measured by multiple methods. To compare lot variation across methods, FA2, FA2G1, and FA2G2 data points from all species were normalized to the most abundant lot and results from MS-based methods were compared to those from FLD, a recent study found that these were the predominant glycoforms across 157 approved products [2]. This assessment resulted in the observation of linear trends for all three comparisons (Fig. 7a). Intact results were included in this comparison by calculating FLD results under the assumption that glycan occupancy is probabilistic [29]. For intact the *R*^2^ value was approximately 0.9. The *R*^2^ value for MAM was lower (0.77) due to an outlier datapoint. Without this datapoint, the *R*^2^ value for MAM was also approximately 0.9. Lower abundance glycans had more discrepancies between methods, and similar trends were not observed. These results indicated that measurements of lot-to-lot changes in high abundance glycans were consistent across methods, but measurements of glycans at lower abundance levels were too variable for statistically significant trends to be observed. However, similar patterns across lots were visually observed between FLD and MAM data for lower abundance glycans, particularly for product B2. The precision of individual methods was also assessed by plotting the %CV for technical replicates of individual glycan measurements against their relative abundance. Results are shown in Fig. 7b; the three methods show comparable precision for high abundance glycans with an increase in %CV at lower abundances. FLD offered the best precision for glycans with abundances less than 0.1%, whereas %CVs for some MAM glycans increased to >20%, these low abundance species were below the LOQ for intact analysis.

**Fig. 8:**
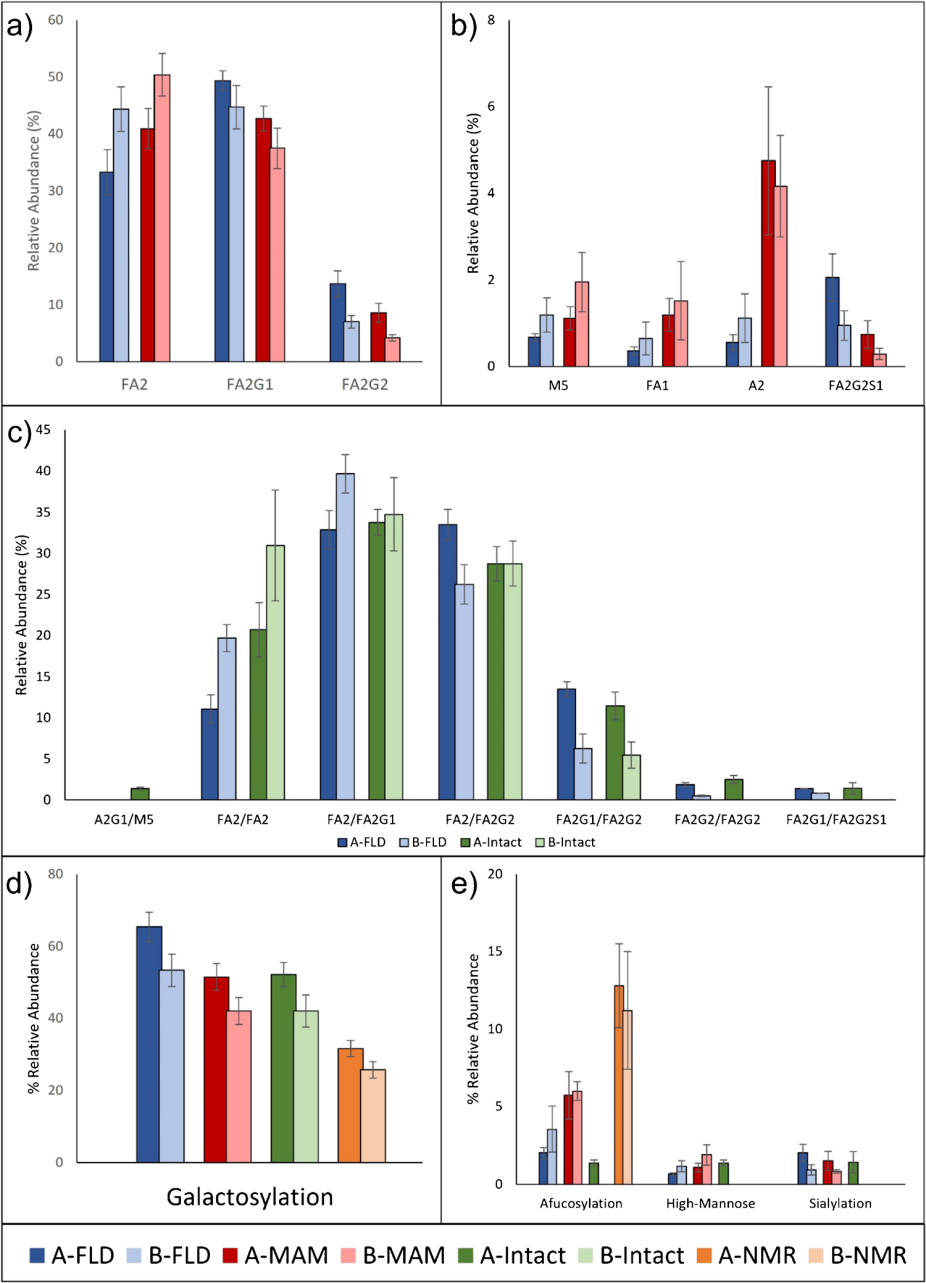
Comparative method analysis. **a**–**b** Released glycan and glycopeptide level measurements. FLD and MAM, were compared. **c** Intact measurements were compared to probabilistic FLD calculated intensities. **d**–**e** All approaches were compared for overarching PQA measurements. Error bars represent standard deviation

The average abundance values of glycan species for individual products were compared between the methods. The product-specific comparisons for the three methods that can identify single glycan species are shown in Fig. 8a. Intact analysis was compared to FLD by calculating FLD glycan pairings under the assumption that glycan occupancy is probabilistic (Fig. 8c) [29]. Both mass spectrometry-based methods measured galactosylated species at a lower abundance than FLD. This finding was consistent with other studies and may be caused by source fragmentation or differing ionization efficiencies of the glycans [30]. While there were differences in absolute values, methods could identify the differences between products for major species. Product A had a lower proportion of FA2 and a higher proportion of FA2G1/FA2G2. FLD and MAM were also in agreement on the relative abundances of M5, FA1, and FA2G2S1. However, the low abundances of these species prevented product-specific comparisons using the other methods.

Product-specific comparisons were also made for overarching PQAs, where glycans were grouped by classifications that may impact mAb safety or efficacy (Table 1). Figure 8d and e show the normalized relative abundance values for each PQA (Table 1). Galactosylation was the most abundant PQA across all methods, with measurements ranging from 31 to 65%. Galactosylation measurements also had the lowest CVs across all methods. A comparison of means found the difference in terminally galactosylated species between products to be statistically significant (*p* <0.05) across all methods. The other PQAs either could not be detected by at least one method, did not agree between methods, or were not found to be different between products. The low abundance of these glycans relative to the other PQAs is likely responsible for these results. Afucosylation was calculated as the sum of six glycan species using MAM and two species for FLD and intact analysis. Sialylation was measured higher in product A using FLD, MAM, and intact methods. MAM was the only method that identified more than one sialylated species. Sialylation was not detected using NMR, which is consistent with the LOD for NMR and the abundance measured in other methods. These approaches are often complementary and may need to be used together to achieve a full picture of the glycan profile. Method selection may be fit-for-purpose, where one method may be superior to another for a specific product based on which glycan patterns are CQAs for that product.

## Conclusions

Within this study, four orthogonal analytical approaches were used to assess the glycan content of nine total lots of rituximab across two drug products. These approaches included multiple high-resolution techniques that were compared to the conventional FLD method. In general, the lower mass analytes (e.g., released glycans and glycopeptides) resulted in increased sensitivity and number of species identified as these species were easier to resolve. However, the higher mass analytes (e.g., NMR and intact MS) offered simplified sample preparation with decreased opportunity for artifacts.

The total number of glycan species identified by each method varied by technique, where the largest number was identified using MAM, and the fewest were identified with intact MS analysis. Additional information was gained from the use of orthogonal methods: intact MS analysis improved the understanding of mAb glycosylation states at the molecular level, while MAM allowed for additional glycan identifications and quantitation of the deglycosylated peptide. Additionally, the use of MS allowed for confirmation of species identifications. FLD, however, was the only method that was able to determine branching patterns, though in cases with lower signal-to-noise this identification may also be achievable by NMR.

The method with the lowest LOQ was MAM (0.1%,), followed by FLD (0.2%); LOQs for intact (1–2%) and NMR (1.5%) were higher. Method precision was comparable for high abundance analytes; however, FLD had the highest precision for low abundance species. A previous study found that MS had better precision for low-abundance analytes when comparing released derivatized glycans quantified by FLD and LC-MS [31].

Lot-to-lot comparisons of the most abundant glycan species showed a linear trend across methods. Relative abundance comparisons between products demonstrated agreement in high abundance glycan levels between the MS-based methods and agreed with the conventional FLD method. Levels of lower abundance glycans were more variable across methods. When glycan PQAs were compared, all methods agreed on the relative galactosylation levels between the two products.

Overall, while MAM was found to be the most sensitive and identified the most species, these methods are complementary and can be used in conjunction to achieve a comprehensive view of the glycosylation state of a mAb. Alternatively, specific methods could be used in a more targeted approach depending on the application. The use of different methods can be tailored based on the specific product being analyzed as well as the process stage where the method is being used.

## Supplementary Information

Below is the link to the electronic supplementary material.

Supplementary file1 (DOCX 786 KB)

## Abbreviations

(U)HPLC: (Ultra) High-performance liquid chromatography
2-AB: 2-Aminobenzamide
ACN: Acetonitrile
CQA: Critical quality attribute
CV: Coefficient of variation
Da: Daltons
DTT: Dithiothreitol
FID: Free induction decay
FLD: Fluorescence detection
HILIC: Hydrophilic interaction liquid chromatography
HRMS: High-resolution mass spectrometry
HSQC: Heteronuclear single quantum coherence
LOD: Limit of detection
mAb: Monoclonal antibody
MAM: Multi-attribute method
MWCO: Molecular weight cutoff
NMR: Nuclear magnetic resonance
PQA: Product quality attribute
QbD: Quality by design
QC: Quality control
SPE: Solid-phase extraction
TCEP: Tris(2-carboxyethyl)phosphine
TSP: Trimethylsilylpropanoic acid
