# Supplementary material for: Assessment of monoclonal antibody glycosylation: a comparative study using HRMS, NMR, and HILIC-FLD
Source: Anal Bioanal Chem. 2024 Apr 6;416(13):3127–37. doi: 10.1007/s00216-024-05261-5 (PMC11541336; doi:10.1007/s00216-024-05261-5)
Supplement: Supplementary file 1 — Supplementary file1 (DOCX 786 KB) [file 216_2024_5261_MOESM1_ESM.docx]

**Supplementary Information**

**Table of Contents**

Table S1 HILIC-UHPLC Parameters

Table S2 MAM LC-MS Parameters

Table S3 Intact LC-MS Parameters

Table S4 Glycan structures

Figure S1 Example HILIC-UHPLC Chromatograms

Table S5 HILIC-FLD Results

Figure S2 Example MAM Chromatograms

Table S6 Peptide Sequences Identified by MAM analysis

Table S7 MAM LC-MS Results

Figure S3 Intact deconvoluted spectra

Table S8 Intact Mass Results

Figure S4 Representative 1H-13C HSQC spectra

Table S9 HSQC Results

Appendix S1 Preparation of Samples for HILIC-FLD analysis

**Table S1.** HILIC-UHPLC Parameters

| **Parameter** | | **Setting** | |
| --- | --- | --- | --- |
| Column | | Waters Acquity BEH Amide cloumn, 2.1 × 150 mm, 1.7 µm, 130 Å | |
| Injection volume | | 10.0 µL | |
| Column temperature | | 60 ^0^C | |
| Auto-sampler temperature | | 10 ^0^C | |
| Mobile Phase A | | 100 mM Ammonium Formate, pH 4.5 | |
| Mobile Phase B | | 100% Acetonitrile | |
| Detector | | FLD | |
| Excitation λ | | 330 nm | |
| Emission λ | | 420 nm | |
| Total run time | | 47.5 min | |
| HPLC Gradient | | | |
| **Time (min)** | **% Mobile Phase A** | **% Mobile Phase B** | **Flow (mL / min)** |
| 0 | 25 | 75 | 0.50 |
| 31.5 | 35.2 | 64.8 | 0.50 |
| 32.0 | 100 | 0 | 0.25 |
| 35.0 | 100 | 0 | 0.25 |
| 36.0 | 25 | 75 | 0.25 |
| 40.0 | 25 | 75 | 0.25 |
| 41.0 | 25 | 75 | 0.50 |
| 47.5 | 25 | 75 | 0.50 |

**Table S2.** MAM LC-MS Parameters

| Parameter |  |  |  |
| --- | --- | --- | --- |
| Column | Agilent Zorbax RRHD StableBond C18  300 Å, 2.1 mm x 150 mm, 1.8 µm | |  |
| Flow Rate | 250 uL/min |  |  |
| Column Temperature | 50 °C |  |  |
| Injection Volume | 5 uL |  |  |
| Mobile Phase A | 0.1% formic acid in water | |  |
| Mobile Phase B | 0.1% formic acid in acetonitrile | |  |
| Gradient |  | |  |
| Time | % Mobile Phase A | % Mobile Phase B | Divert Valve |
| 0 | 99 | 1 | MS |
| 5 | 99 | 1 | MS |
| 6 | 90 | 10 | MS |
| 70 | 65 | 35 | MS |
| 72 | 10 | 90 | Waste |
| 77 | 10 | 90 | Waste |
| 77.1 | 99 | 1 | Waste |
| 90 | 99 | 1 | Waste |
|  |  |  |  |
| LC System | Thermo Accela |  |  |
| MS System | Thermo Q-Exactive Quadrupole-Orbitrap |  |  |
| Spray Voltage | 3.5 kV |  |  |
| Capillary Temperature | 250 C |  |  |
| MS Mode | Positive |  |  |
| m/z Range | 300 – 1800 |  |  |

**Table S3.** Intact and reduced LC-MS Parameters

| Parameter |  |  |
| --- | --- | --- |
| Column | Waters Acquity UPLC Protein BEH C4, 300 Å, 1 mm x 150 mm, 1.7 µm | |
| Flow Rate | 150 uL/min |  |
| Column Temperature | 65°C |  |
| Injection Volume | 5 uL |  |
| Mobile Phase A | 0.1% formic acid in water | |
| Mobile Phase B | 0.1% formic acid in acetonitrile | |
| Gradient |  | |
| Time | % Mobile Phase A | % Mobile Phase B |
| 0 | 95 | 5 |
| 1 | 95 | 5 |
| 2 | 80 | 20 |
| 15 | 40 | 60 |
| 16 | 5 | 95 |
| 18 | 5 | 95 |
| 19 | 95 | 5 |
| 20 | 95 | 5 |
| LC System | Dionex UHPLC 3000 |  |
| MS System | Thermo Orbitrap Fusion |  |
| Spray Voltage | 3.5 kV |  |
| Capillary Temperature | 250 C |  |
| MS Mode | Positive |  |
| m/z Range | 1000-4000 |  |
| Resolution | 15,000 |  |

**Table S4.** Glycan structures with Oxford notation names

| Name | Structure |
| --- | --- |
| De-glycosylated |  |
| A1 | 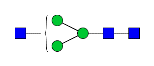 |
| FA1 | 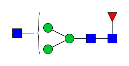 |
| FA1G1 | 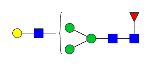 |
| A2 | 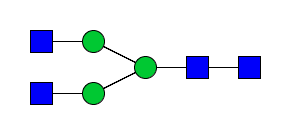 |
| FA2 | 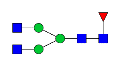 |
| M5 | 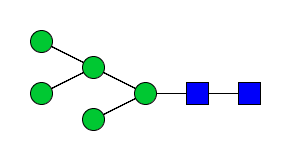 |
| A2[6]G1 | 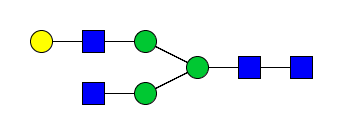 |
| A2[3]G1 | 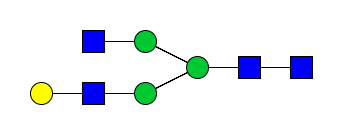 |
| FA2G1 | 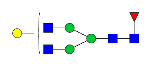 |
| FA2[6]G1 | 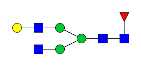 |
| FA2[3]G1 | 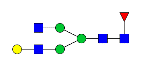 |
| A2G2 | 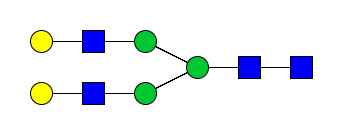 |
| FA2G2 | 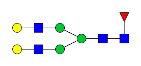 |
| FA2G1S1 | 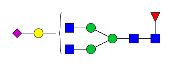 |
| FA2G2S1 | 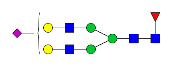 |
| FA2G2S2 | 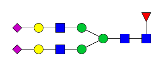 |


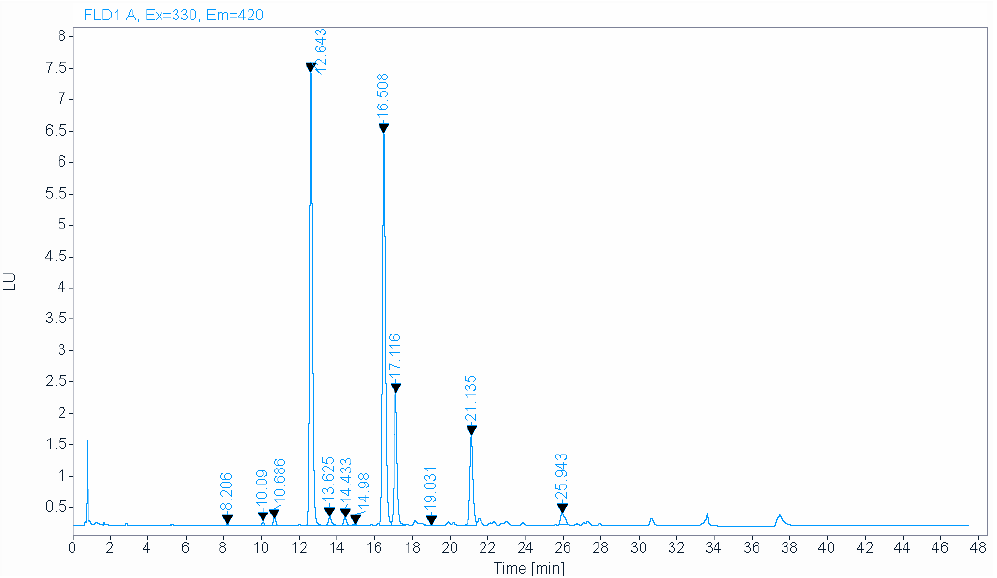


**A1**

**FA2**

**FA2[6]G1**

**A2G2**

**FA2G2**

**FA2G2S1**

**M5**

**FA1**

**A2**

**A2[3]G1**

**FA2[3]G1**

**A2[6]G1**


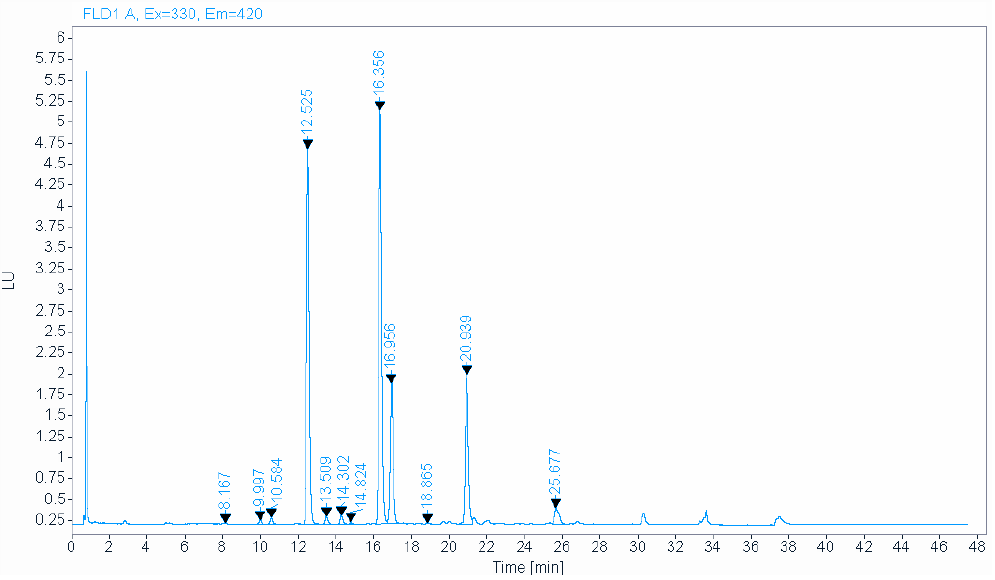


**A1**

**A2**

**FA2**

**A2[3]G1**

**FA2[6]G1**

**A2G2**

**FA2G2**

**FA2G2S1**

**A2[6]G1**

**FA2[3]G1**

**M5**

**FA1**

**Figure S1.** Example HILIC-UHPLC Chromatograms for Product A (Top) and Product B (Bottom)

**Table S5.** Glycan Quantitation with HILIC-FLD Results

|  | M5 | FA1 | A2 | FA2 | A2[3]G1 | A2[6]G1 | FA2[3]G1 | FA2[6]G1 | FA2G2 | FA2G2S1 |
| --- | --- | --- | --- | --- | --- | --- | --- | --- | --- | --- |
|  | Average Fractional Abundance (%) | | | | | | | | | |
| A1 | 0.56 | 0.244 | 0.32 | 28 | <LOQ | 0.723 | 13.3 | 38.2 | 16.4 | 2.1 |
| A2 | 0.72 | 0.50 | 0.654 | 35.5 | <LOQ | 0.83 | 12.4 | 34.9 | 12.6 | 2.0 |
| A3 | 0.60 | 0.27 | 0.45 | 30 | <LOQ | 0.70 | 13.10 | 37.3 | 15.2 | 2.4 |
| A4 | 0.58 | 0.28 | 0.45 | 29.5 | <LOQ | 0.68 | 13.16 | 37.3 | 15.5 | 2.5 |
| A5 | 0.74 | 0.47 | 0.57 | 36 | <LOQ | 0.77 | 12.4 | 35.96 | 11.9 | 1.7 |
| A6 | 0.75 | 0.39 | 0.51 | 33.2 | <LOQ | 0.89 | 12.7 | 35.6 | 13.6 | 2.30 |
| A7 | 0.72 | 0.33 | 0.90 | 39.3 | 0.22 | 0.876 | 12.55 | 34.06 | 9.8 | 1.2 |
| B1 | 0.81 | 0.29 | 0.59 | 41 | <LOQ | 0.69 | 12.4 | 35.5 | 7.8 | 1.2 |
| B2 | 1.52 | 0.98 | 1.61 | 46.9 | 0.23 | 1.66 | 10.7 | 29.8 | 6.0 | 0.63 |
|  | Standard Deviation | | | | | | | | | |
| A1 | 0.02 | 0.009 | 0.01 | 1 | N/A | 0.008 | 0.1 | 0.4 | 0.8 | 0.7 |
| A2 | 0.05 | 0.01 | 0.009 | 0.5 | N/A | 0.01 | 0.1 | 0.3 | 0.4 | 0.6 |
| A3 | 0.04 | 0.01 | 0.02 | 1 | N/A | 0.02 | 0.09 | 0.1 | 0.8 | 0.2 |
| A4 | 0.03 | 0.02 | 0.03 | 0.2 | N/A | 0.03 | 0.07 | 0.3 | 0.2 | 0.2 |
| A5 | 0.03 | 0.02 | 0.02 | 1 | N/A | 0.03 | 0.2 | 0.10 | 0.9 | 0.2 |
| A6 | 0.03 | 0.02 | 0.01 | 0.4 | N/A | 0.02 | 0.3 | 0.2 | 0.4 | 0.09 |
| A7 | 0.02 | 0.02 | 0.03 | 0.8 | 0.01 | 0.002 | 0.06 | 0.06 | 0.7 | 0.2 |
| B1 | 0.07 | 0.03 | 0.06 | 2 | N/A | 0.01 | 0.5 | 0.9 | 0.9 | 0.1 |
| B2 | 0.05 | 0.01 | 0.06 | 0.6 | 0.01 | 0.03 | 0.1 | 0.3 | 0.3 | 0.06 |

|  | M5 | A1 | FA1 | A2 | FA2 | A2[3]G1 | A2[6]G1 | FA2[3]G1 | FA2[6]G1 | A2G2 | FA2G2 | FA2G2S1 |
| --- | --- | --- | --- | --- | --- | --- | --- | --- | --- | --- | --- | --- |
|  | Average Fractional Abundance (%) | | | | | | | | | | | |
| A1 | 0.56 | 0.02 | 0.24 | 0.32 | 28 | 0.10 | 0.72 | 13.3 | 38.1 | 0.026 | 16.4 | 2.1 |
| A2 | 0.71 | 0.07 | 0.50 | 0.65 | 35.4 | 0.147 | 0.82 | 12.3 | 34.8 | 0.03 | 12.5 | 2.0 |
| A3 | 0.60 | 0.05 | 0.27 | 0.45 | 30 | 0.12 | 0.70 | 13.1 | 37.2 | 0.01 | 15.2 | 2.4 |
| A4 | 0.58 | 0.02 | 0.27 | 0.45 | 29.5 | 0.11 | 0.68 | 13.1 | 37.2 | 0.02 | 15.5 | 2.5 |
| A5 | 0.74 | 0.06 | 0.46 | 0.57 | 35 | 0.12 | 0.76 | 12.3 | 35.9 | 0.02 | 11.9 | 1.7 |
| A6 | 0.75 | 0.03 | 0.39 | 0.51 | 33.1 | 0.14 | 0.891 | 12.7 | 35.5 | 0.04 | 13.6 | 2.3 |
| A7 | 0.71 | 0.04 | 0.33 | 0.90 | 39.3 | 0.22 | 0.88 | 12.5 | 34.0 | 0.05 | 9.8 | 1.2 |
| B1 | 0.81 | 0.028 | 0.29 | 0.59 | 41 | 0.13 | 0.68 | 12.4 | 35.4 | 0.016 | 7.8 | 1.2 |
| B2 | 1.52 | 0.10 | 0.98 | 1.60 | 46.8 | 0.23 | 1.66 | 10.7 | 29.7 | 0.01 | 6.0 | 0.63 |
|  | Standard Deviation | | | | | | | | | | | |
| A1 | 0.02 | 0.01 | 0.01 | 0.01 | 1 | 0.01 | 0.01 | 0.1 | 0.4 | 0.005 | 0.8 | 0.7 |
| A2 | 0.05 | 0.02 | 0.01 | 0.01 | 0.5 | 0.005 | 0.02 | 0.1 | 0.4 | 0.02 | 0.3 | 0.6 |
| A3 | 0.04 | 0.03 | 0.01 | 0.02 | 1 | 0.01 | 0.02 | 0.1 | 0.1 | 0.01 | 0.8 | 0.2 |
| A4 | 0.03 | 0.01 | 0.02 | 0.03 | 0.2 | 0.01 | 0.03 | 0.1 | 0.3 | 0.01 | 0.2 | 0.2 |
| A5 | 0.03 | 0.01 | 0.02 | 0.02 | 1 | 0.01 | 0.03 | 0.2 | 0.1 | 0.01 | 0.9 | 0.2 |
| A6 | 0.03 | 0.02 | 0.02 | 0.01 | 0.4 | 0.01 | 0.02 | 0.3 | 0.2 | 0.01 | 0.4 | 0.1 |
| A7 | 0.02 | 0.01 | 0.02 | 0.03 | 0.8 | 0.01 | 0.002 | 0.1 | 0.1 | 0.02 | 0.7 | 0.2 |
| B1 | 0.07 | 0.004 | 0.03 | 0.06 | 2 | 0.01 | 0.01 | 0.5 | 0.9 | 0.002 | 0.9 | 0.1 |
| B2 | 0.05 | 0.02 | 0.01 | 0.06 | 0.6 | 0.01 | 0.03 | 0.1 | 0.3 | 0.01 | 0.3 | 0.06 |


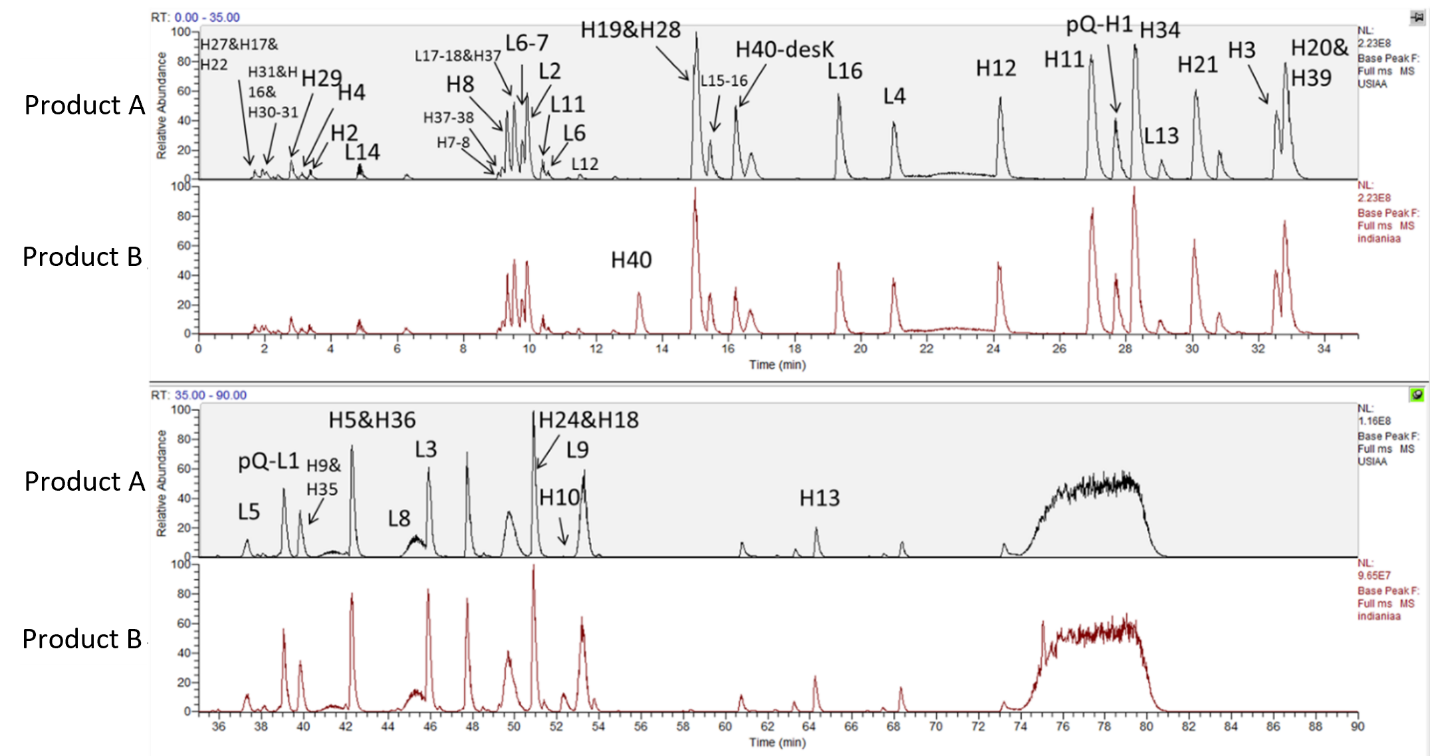


**Figure S2.** MAM TIC of US Product A and Product B with identified tryptic peptides.

**Table S6**: Peptide Sequences Identified by MAM analysis

| **Label** | **Peptide sequence** | **Start** | **End** | **Modification State** | **Theoretical monoisotopic mass (uncharged)** |
| --- | --- | --- | --- | --- | --- |
| pQ-H1 | QVQLQQPGAELVKPGASVK | 1 | 19 | Gln1(Pyro-Glu) | 1959.0790 |
| H2 | MSCK | 20 | 23 | C22(Carboxymethyl) | 525.1927 |
| H3 | ASGYTFTSYNMHWVK | 24 | 38 |  | 1790.8086 |
| H4 | QTPGR | 39 | 43 |  | 557.2921 |
| H5 | GLEWIGAIYPGNGDTSYNQK | 44 | 63 |  | 2182.0331 |
| H7-8 | GKATLTADK | 66 | 74 |  | 718.3861 |
| H8 | ATLTADK | 68 | 74 |  | 718.3861 |
| H9 | SSSTAYMQLSSLTSEDSAVYYCAR | 75 | 98 | C96(Carboxymethyl) | 2677.1524 |
| H10 | STYYGGDWYFNVWGAGTTVTVSAASTK | 99 | 125 |  | 2888.3293 |
| H11 | GPSVFPLAPSSK | 126 | 137 |  | 1185.6394 |
| H12 | STSGGTAALGCLVK | 138 | 151 | C148(Carboxymethyl) | 1321.6548 |
| H13 | DYFPEPVTVSWNSGALTSGVHTFPAVLQSSGLYSLSSVVTVPSSSLGTQTYICNVNHKPSNTK | 152 | 214 | C204(Carboxymethyl) | 6713.2912 |
| H16 | AEPK | 219 | 222 |  | 443.2380 |
| H17 | SCDK | 223 | 226 | C224(Carboxymethyl) | 509.1792 |
| H18 | THTCPPCPAPELLGGPSVFLFPPKPK | 227 | 252 | C230(Carboxymethyl), C233(Carboxymethyl) | 2845.4183 |
| H19 | DTLMISR | 253 | 259 |  | 834.4269 |
| H20 | TPEVTCVVVDVSHEDPEVK | 260 | 278 | C265(Carboxymethyl) | 2139.0042 |
| H21 | FNWYVDGVEVHNAK | 279 | 292 |  | 1676.7947 |
| H22 | TKPR | 293 | 296 |  | 500.3071 |
| H24 | VVSVLTVLHQDWLNGK | 306 | 321 |  | 1806.9992 |
| H27 | VSNK | 327 | 330 |  | 446.2489 |
| H28 | ALPAPIEK | 331 | 338 |  | 837.4960 |
| H29 | TISK | 339 | 342 |  | 447.2693 |
| H31 | GQPR | 345 | 348 |  | 456.2445 |
| H30-31 | AKGQPR | 343 | 348 |  | 655.3765 |
| H34 | NQVSLTCLVK | 365 | 374 | C371(Carboxymethyl) | 1161.6064 |
| H35 | GFYPSDIAVEWESNGQPENNYK | 375 | 396 |  | 2543.1241 |
| H36 | TTPPVLDSDGSFFLYSK | 397 | 413 |  | 1872.9145 |
| H37 | LTVDK | 414 | 418 |  | 574.3326 |
| H37-38 | LTVDKSR | 414 | 420 |  | 817.4657 |
| H39 | WQQGNVFSCSVMHEALHNHYTQK | 421 | 443 | C429(Carboxymethyl) | 2801.2438 |
| H40 | SLSLSPGK | 444 | 451 |  | 787.4439 |
| H40-desK | SLSLSPG | 444 | 450 |  | 659.3490 |
| pQ-L1 | QIVLSQSPAILSASPGEK | 1 | 18 | Gln1(Pyro-Glu) | 1806.9728 |
| L2 | VTMTCR | 19 | 24 | C23(Carboxymethyl) | 767.3306 |
| L3 | ASSSVSYIHWFQQKPGSSPKPWIYATSNLASGVPVR | 25 | 60 |  | 3932.0009 |
| L4 | FSGSGSGTSYSLTISR | 61 | 76 |  | 1605.7634 |
| L5 | VEAEDAATYYCQQWTSNPPTFGGGTK | 77 | 102 | C87(Carboxymethyl) | 2878.2392 |
| L6 | LEIK | 103 | 106 |  | 501.3162 |
| L6-7 | LEIKR |  |  |  | 657.4173 |
| L8 | TVAAPSVFIFPPSDEQLK | 108 | 125 |  | 1945.0197 |
| L9 | SGTASVVCLLNNFYPR | 126 | 141 | C133(Carboxymethyl) | 1797.8720 |
| L11 | VQWK | 145 | 148 |  | 559.3118 |
| L12 | VDNALQSGNSQESVTEQDSK | 149 | 168 |  | 2134.9614 |
| L13 | DSTYSLSSTLTLSK | 169 | 182 |  | 1501.7511 |
| L14 | ADYEK | 183 | 187 |  | 624.2755 |
| L16 | VYACEVTHQGLSSPVTK | 190 | 206 | C193(Carboxymethyl) | 1875.9037 |
| L15-16 | HKVYACEVTHQGLSSPVTK | 188 | 206 | C(Carboxymethyl) | 2141.0575 |
| L17-18 | SFNRGEC | 207 | 213 | C(Carboxymethyl) | 869.3338 |

**Table S7.** Glycan quantitation with MAM LC-MS Results

|  | degly | M5 | FA1 | A2 | FA1G1 | FA2 | FA2G1 | FA2G2 | FA2G1S1 | FA2G2S1 | FA2G2S2 |
| --- | --- | --- | --- | --- | --- | --- | --- | --- | --- | --- | --- |
|  | Average Fractional Abundance (%) | | | | | | | | | | |
| A1 | 0.74 | 0.99 | 1.11 | 4.45 | 0.66 | 34.89 | 44.05 | 10.92 | 0.27 | 1.13 | 0.79 |
| A2 | 0.75 | 1.28 | 1.56 | 3.29 | 0.75 | 41.75 | 40.36 | 8.26 | 0.31 | 0.91 | 0.78 |
| A3 | 0.71 | 0.87 | 1.13 | 4.08 | 0.55 | 36.9 | 43.98 | 9.88 | 0.27 | 0.97 | 0.67 |
| A4 | 0.74 | 0.64 | 0.37 | 8.43 | 0.22 | 39.8 | 43.07 | 6.37 | <LOQ | 0.23 | 0.12 |
| A5 | 0.65 | 1.25 | 1.43 | 4.12 | 0.61 | 41.24 | 41.07 | 8.27 | 0.18 | 0.61 | 0.56 |
| A6 | 0.85 | 1.34 | 1.35 | 4.17 | 0.77 | 39.92 | 41.22 | 8.64 | 0.24 | 0.8 | 0.69 |
| A7 | 1.02 | 1.23 | 1.21 | 4.06 | 0.55 | 45.71 | 38.68 | 6.42 | 0.17 | 0.39 | 0.55 |
| B1 | 0.63 | 1.32 | 0.69 | 5.13 | 0.23 | 46.59 | 40.02 | 4.57 | 0.2 | 0.4 | 0.23 |
| B2 | 0.42 | 2.5 | 2.28 | 3.05 | 1.44 | 52.22 | 33.54 | 3.69 | 0.17 | 0.17 | 0.52 |
|  | Standard Deviation | | | | | | | | | | |
| A1 | 0.06 | 0.07 | 0.13 | 0.65 | 0.08 | 0.28 | 0.41 | 0.23 | 0.04 | 0.04 | 0.05 |
| A2 | 0.04 | 0.05 | 0.06 | 0.75 | 0.07 | 1.47 | 1.35 | 0.42 | 0.02 | 0.08 | 0.03 |
| A3 | 0.02 | 0.06 | 0.08 | 0.52 | 0.05 | 1.63 | 1.41 | 0.53 | 0.04 | 0.08 | 0.03 |
| A4 | 0.05 | 0.25 | 0.08 | 0.55 | 0.04 | 1.59 | 1.54 | 0.37 | N/A | 0.06 | 0.07 |
| A5 | 0.03 | 0.03 | 0.09 | 1.13 | 0.03 | 0.71 | 1.08 | 0.63 | 0.02 | 0.08 | 0.03 |
| A6 | 0.04 | 0.05 | 0.10 | 0.33 | 0.02 | 1.71 | 1.21 | 0.55 | 0.04 | 0.11 | 0.11 |
| A7 | 0.08 | 0.08 | 0.07 | 0.39 | 0.10 | 1.39 | 1.08 | 0.35 | 0.02 | 0.08 | 0.04 |
| B1 | 0.00 | 0.18 | 0.11 | 0.35 | 0.08 | 1.80 | 1.40 | 0.28 | 0.06 | 0.03 | 0.05 |
| B2 | 0.01 | 0.05 | 0.09 | 0.28 | 0.04 | 1.00 | 1.07 | 0.26 | 0.11 | 0.03 | 0.10 |


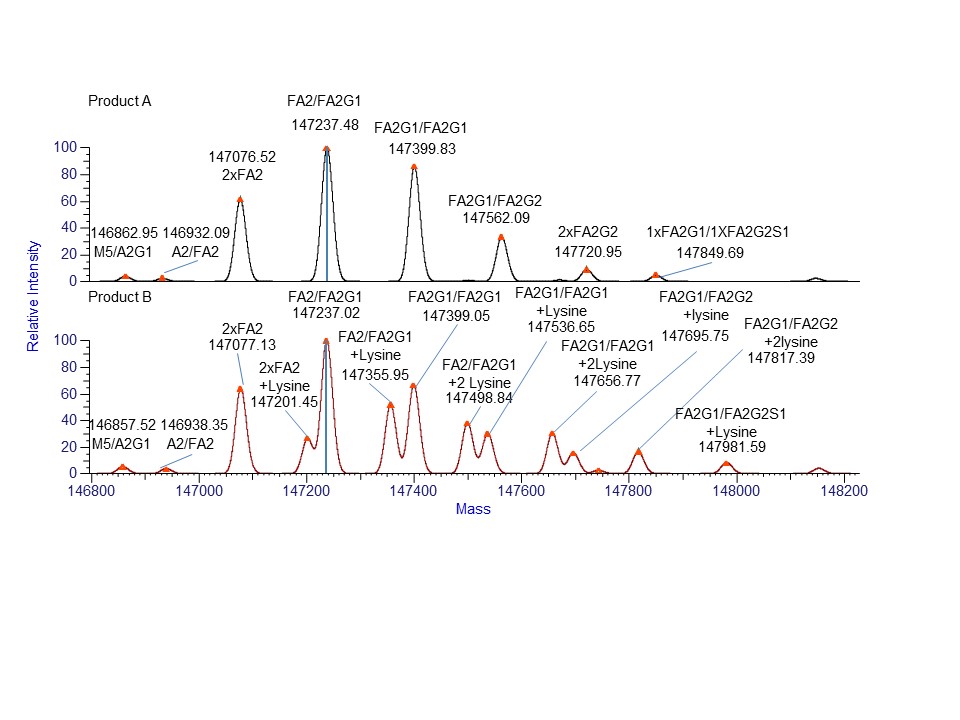


**Figure S3.** Intact deconvoluted spectra

**Table S8.** Glycan Quantitation using LC-MS Intact Mass Analysis Results

|  | A2G1/  M5 | FA2/  FA2 | FA2/  FA2G1 | FA2/  FA2G2 | FA2G1/  FA2G2 | FA2G2/  FA2G2 | FA2G1/  FA2G2S1 |
| --- | --- | --- | --- | --- | --- | --- | --- |
|  | Average Fractional Abundance (%) | | | | | | |
| A1 | 1.22 | 16.2 | 32.1 | 31.2 | 13.7 | 3.2 | 2.1 |
| A2 | 1.60 | 23.2 | 33.2 | 27.0 | 10.4 | 2.3 | 1.5 |
| A3 | 1.19 | 17.4 | 32.8 | 30.6 | 12.9 | 2.7 | 1.8 |
| A4 | 1.2 | 18.4 | 32.9 | 29.9 | 12.5 | 2.7 | 1.9 |
| A5 | 1.5 | 22.0 | 33.1 | 28.4 | 11.0 | 2.3 | 1.2 |
| A6 | 1.4 | 20.8 | 34.2 | 28.0 | 10.9 | 2.5 | 1.4 |
| A7 | 1.43 | 26.2 | 37.1 | 25.0 | 8.4 | 1.8 | <LOQ |
| B1 | <LOQ | 24 | 37 | 29.8 | 6.6 | ND | <LOQ |
| B2 | <LOQ | 37 | 31 | 26 | 4.1 | ND | <LOQ |
|  | Standard Deviation | | | | | | |
| A1 | 0.09 | 0.2 | 0.1 | 0.3 | 0.3 | 0.2 | 0.2 |
| A2 | 0.07 | 0.2 | 0.4 | 0.3 | 0.2 | 0.2 | 0.2 |
| A3 | 0.09 | 0.4 | 0.2 | 0.5 | 0.3 | 0.3 | 0.2 |
| A4 | 0.1 | 0.3 | 0.6 | 0.5 | 0.2 | 0.2 | 0.2 |
| A5 | 0.2 | 0.3 | 0.5 | 0.5 | 0.2 | 0.3 | 0.3 |
| A6 | 0.2 | 0.4 | 0.5 | 0.3 | 0.2 | 0.2 | 0.3 |
| A7 | 0.09 | 0.7 | 0.4 | 0.6 | 0.4 | 0.3 | NA |
| B1 | NA | 1 | 2 | 2 | 0.7 | NA | NA |
| B2 | NA | 3 | 4 | 2 | 1 | NA | NA |

**
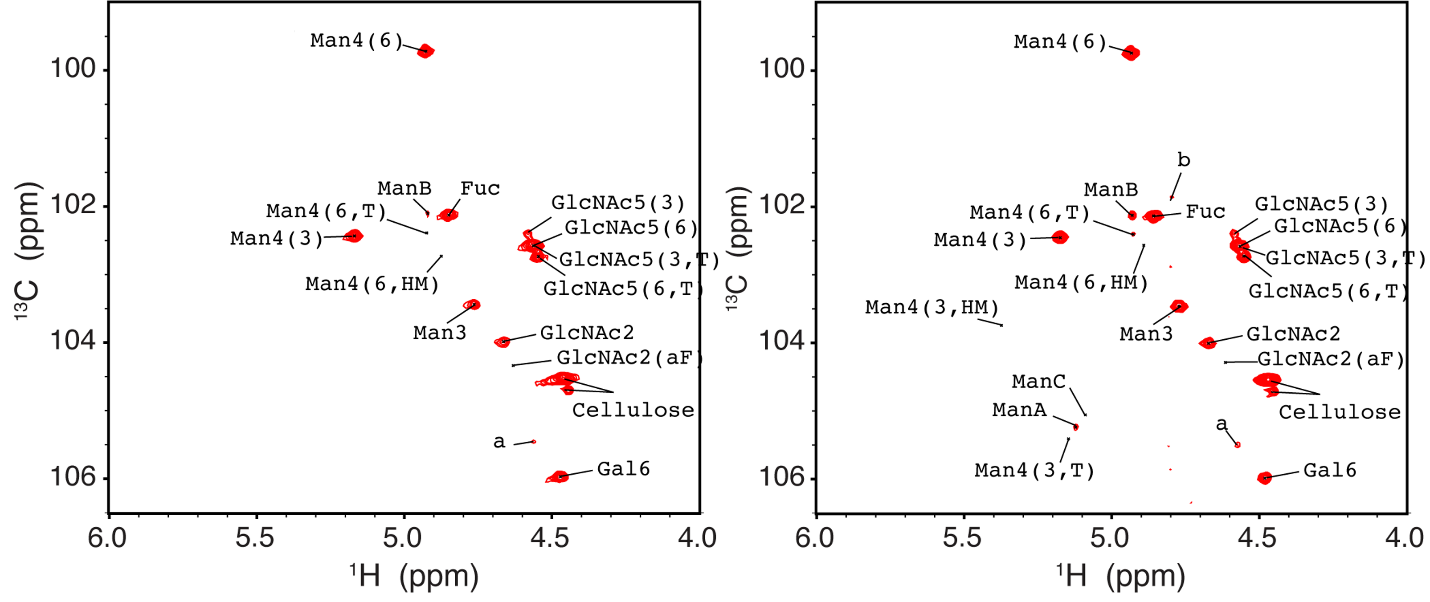
**

**Figure S4.** The representative 1H-13C HSQC spectra of rituximab from Product A (left) and Product B (right). The shown spectrum was from the samples of lot 3209283 and RIBV3412. The plotting threshold was set to S/N = 5. Peaks of Man4(3,T), Man4(3,HM), ManA (mannose at position 5 of 1-6/1-3branch) and ManC (mannose at position 5 of 1-3 branch) were not observed in product A, suggesting less mannose species in product A.

**Table S9.** Glycan analysis (%) by HSQC peak intensities for rituximab Fc fragment.

| Drug Product | | Total  Afucosylation | High-mannose  (M4-M9) | Galactosylation^a^ |
| --- | --- | --- | --- | --- |
| Equation | | 1 | 2 | 3 |
| Product A | A1 | 15.5 | 3.79 | 35.2 |
|  | A2 | 11.6 | NA^b^ | 30.0 |
|  | A3 | 14.4 | NA^b^ | 32.4 |
|  | A4 | NA^b^ | NA^b^ | 33.6 |
|  | A5 | NA^b^ | NA^b^ | 29.9 |
|  | A6 | NA^b^ | NA^b^ | 31.4 |
|  | A7 | 9.65 | NA^b^ | 28.9 |
|  | ***Average*** | ***12.8*** | ***3.8*** | ***31.6*** |
|  | ***STDV*** | ***2.7*** | ***-*** | ***2.2*** |
|  | ***CV%*** | ***21*** | ***-*** | ***7*** |
| Product B | B1 | 8.42 | NA^b^ | 27.3 |
|  | B2 | 13.9 | 4.93 | 24.1 |
|  | ***Average*** | ***11.2*** | ***4.9*** | ***25.7*** |
|  | ***STDV*** | ***3.9*** | ***-*** | ***2.3*** |
|  | ***CV%*** | ***35*** | ***-*** | ***9*** |

^a^ 100% galactosylation here is two galactoses in one complex N-glycan, *e.g.*, (F)A2G2.

^b^ Not available due to low signal to noise ratio. The signal to noise cut-off is S/N >=3.

**Appendix S1: Preparation of Samples for HILIC-FLD analysis**

**Centrifugal Filter Preparation**

1. Equilibrate the filter (10KD, PALL Corporation, Ref OD010C33, Lot FE3150) by adding 100 µL of digestion buffer.
2. Centrifuge at 10000 × g for 3 min.
3. Add another 100 µL of digestion buffer to the filter.
4. Add 20 µL of rituximab sample (equivalent to 200 µg) to the filter.
5. Centrifuge at 13000 × g for 2 min.
6. Discard the flow through.
7. Wash the filter with 350 µL of digestion buffer.
8. Centrifuge at 13000 × g for 5 minutes.
9. Discard the flow through.
10. Repeat steps 7 – 8.
11. Discard the flow through and use a new collection tube.

**De-Glycosylation**

1. Add 48 µL of digestion buffer and 2 µL of PNGase F.
2. Incubate samples in a heating block at 45 °C for 1 hour while mixing at 500 rpm.
3. Cool samples to room temperature.
4. Centrifuge at 14000 × g for 5 min, to collect the *N*-glycans in the collection tube.
5. To the collection tube, add 5 µL of 200 mmol/L acetic acid.
6. Vortex and spin down.
7. Incubate for 15 minutes at 45 °C while mixing at 500 rpm.
8. Cool to room temperature.

**Labeling**

1. Add 10 µL of labeling reagent (5 µL of 2-AB solution and 5 µL of Reductant solution)
2. Vortex. Spin down briefly.
3. Cap the tubes tightly and incubate for 2 hours at 65 °C in a heating block at 500 rpm.
4. Cool samples to room temperature.
5. Spin down.

**Removal of excess labeling reagent**

1. Add 1 mL of 96% acetonitrile to the labeled glycans and mix.
2. Rinse required number of cartridges with 1 mL of water under low vacuum.
3. Rinse cartridges with 1 mL of 96% acetonitrile under low vacuum or gravity.
4. Apply each labeled glycan mixture to individual cartridges.
5. Let sample flow through by gravity.
6. Wash cartridges 3x with 1.0 mL of 96% acetonitrile under gravity.
7. After, 3^rd^ washing, put the cartridges into 1.5 µL tubes and do lower speed centrifuging at 1000 × g for 1 min to completely dry the cartridge.
8. Transfer the cartridges into a new 1.5 mL tube.
9. Add 100 µL of 20% acetonitrile and allow to absorb into the cartridge.
10. Elute the labeled glycans by centrifuging briefly at 2000 × g for 2 min.
11. Mix samples 1:1 with 96% acetonitrile (100 µL) prior to HPLC analysis.
12. Vortex, spin down and transfer the sample into a HPLC vial.
